# Unusual Presentation of Cardiac Myxoma Mimicking Chronic Obstructive Pulmonary Disease (COPD) Exacerbation: A Report of a Rare Case

**DOI:** 10.7759/cureus.61967

**Published:** 2024-06-08

**Authors:** Jorge Pimentel, Gabriela Suero Taveras, Alessia Floriani Alvarez

**Affiliations:** 1 Internal Medicine, CEDIMAT (Centros de Diagnóstico, Medicina Avanzada y Telemedicina), Santo Domingo, DOM; 2 Medicine, Instituto Tecnológico de Santo Domingo (INTEC), Santo Domingo, DOM

**Keywords:** myxoma, right atrial myxoma, heart, cardiology, surgical replacement of the valve

## Abstract

A cardiac myxoma is an authentic tumor that develops within the heart. Despite the typically benign histological characteristics, a cardiac myxoma may, on occasion, exhibit behavior reminiscent of malignant tumors. Most of these myxomas localize in the left atrium, often originating from a stalk near the foramen ovale region. The conventional presentation of cardiac myxomas includes a combination of obstruction, clot formation, and systemic symptoms, mirroring various other prevalent systemic diseases. They may manifest either spontaneously or through hereditary transmission. While familial myxomas are commonly linked to discernible genetic mutations, the precise molecular mechanisms underlying spontaneous myxomas remain somewhat enigmatic. Many individuals with myxomas may remain asymptomatic. However, should symptoms manifest, they can prove nonspecific and pose challenges in interpretation, particularly in instances of spontaneous heart myxomas. This report describes a 58-year-old female patient who presented with increasing severity of exertional dyspnea over a six-month duration. Initial differential diagnoses included common pulmonary and cardiac conditions, with a primary focus on chronic obstructive pulmonary disease and congestive heart failure. An echocardiogram revealed a large mass in the left atrium suggestive of a cardiac myxoma. Surgical resection confirmed the diagnosis. This case underscores the significance of including cardiac myxoma in differential diagnoses for progressive exertional dyspnea. Early detection and surgical intervention are crucial in mitigating potential complications like stroke, heart failure, or sudden cardiac death.

## Introduction

Cardiac myxoma, a rare benign cardiac tumor, is primarily found in the atria of the heart. Its rarity often leads to it being overshadowed by other prevalent cardiac and pulmonary conditions in clinical diagnoses. Despite its benign nature, this tumor poses considerable risks due to its propensity to cause obstructions or embolisms, which can result in severe complications if left undetected or untreated [1-5].

Many patients with cardiac myxoma remain asymptomatic, allowing the tumor to grow unnoticed for extended periods. When symptoms do present, they can be diverse, often depending on factors such as the tumor's size, precise location within the heart, and the extent to which it might have caused obstructions or developed embolisms [6-8]. Some common symptoms include fatigue, palpitations, chest pain, or syncope [9,10]. However, in some cases, it may manifest as progressive exertional dyspnea [11]. Common pulmonary conditions like chronic obstructive pulmonary disease (COPD) can be part of the differential diagnoses, given their prevalence and symptomatology [12]. Additionally, congestive heart failure (CHF) can also be a leading consideration due to the overlapping symptoms of dyspnea on exertion [13].

A single-centered study in China revealed that solid myxomas were associated with more arrhythmias, a larger tumor volume, implantation in the interatrial septum, and a need for concomitant surgery compared with papillary myxomas [14]. Further studies should determine whether serum or histological markers could be routinely used in combination with echocardiograms, magnetic resonance imaging, and computed tomography for the prediction of recurrent myxomas during annual follow-up examinations. If left undetected or untreated, cardiac myxomas can lead to catastrophic events like strokes, heart failure, or even sudden cardiac death. Thus, maintaining a comprehensive diagnostic perspective is essential to ensure optimal patient outcomes [15].

In this report, we describe the case of a 55-year-old female patient with cardiac myxoma which manifested as progressive exertional dyspnea and differential diagnoses included COPD and CHF. This case serves as a poignant reminder to clinicians about the significance of including cardiac myxoma in differential diagnoses, especially when patients present with progressive exertional dyspnea. The early detection and subsequent surgical intervention in this case played a pivotal role in averting potential life-threatening complications.

## Case presentation

A 55-year-old female patient presented with exertional dyspnea for the past seven months upon walking more than a block, which had recently worsened, preventing her from speaking when walking even short distances. Dyspnea improved with rest. Additionally, she reported oppressive chest pain, not related to exertion, which had begun with the onset of dyspnea. Her medical history included preeclampsia and gestational diabetes in 2002, both resolved, and systemic arterial hypertension since approximately 2008. The patient had a history of asthma, well controlled with salbutamol as needed. Regarding her pharmacological treatment, she was on amlodipine 5 mg and furosemide 40 mg daily. She reported no allergies, toxicities, or relevant family history. It is important to note that the patient did not smoke.

The patient presented with a blood pressure of 130/80 mmHg, a heart rate of 106 beats per minute, an oxygen saturation of 95% in room air, weighed 65.8 kg, and measured 159 cm, with a BMI of 26 kg/m^2^. She did not present with jugular venous distension, hepatojugular reflux, or carotid bruits. Her heart sounds were regular with a grade II/VI mid-systolic murmur in the left parasternal region that decreased upon sitting and a proto-diastolic plop. The lungs were well-ventilated without crackles, and the abdomen had adequate peristalsis without anomalies. The limbs showed no edema and had adequate peripheral pulses on both sides.

**Figure 1:**
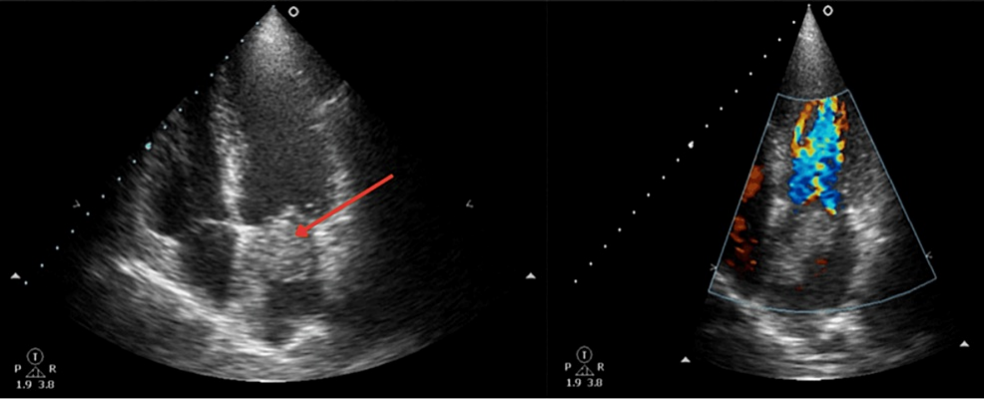
Echocardiogram detects an intracardiac mass in the left atrium

**Figure 2:**
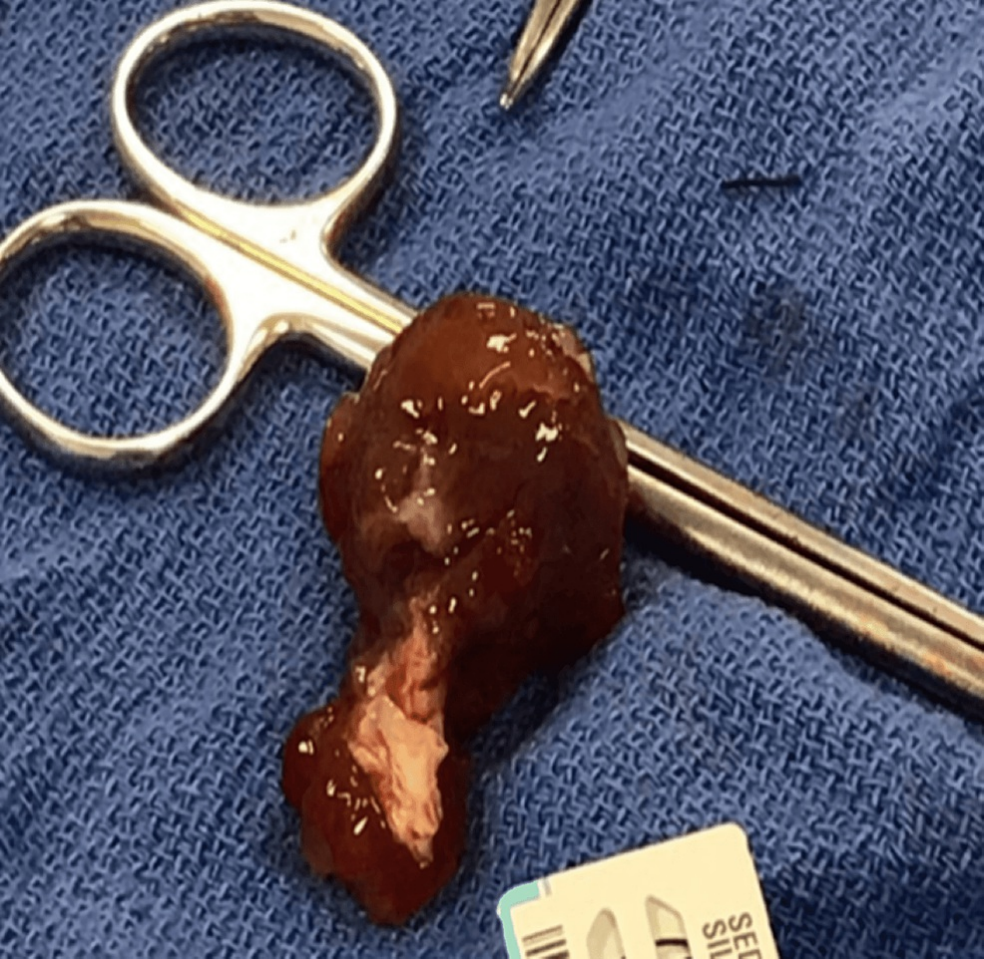
Myxoma mass

Various tests were conducted, including a complete blood count, chemistry, urine analysis, blood typing, viral panel, chest x-ray, electrocardiogram, and echocardiogram. Most of the results were found to be normal with a blood type of O Rh negative. However, the electrocardiogram revealed regular sinus tachycardia, with growth in both atria. The echocardiogram detected an intracardiac mass in the left atrium (Figure 1), likely a myxoma, with an ejection fraction of 56.5%. A coronary angiography was indicated, and surgery was scheduled to resect the mass. Subsequently, a sternotomy and other surgical procedures were carried out to resect the possible myxoma mass (Figure 2).

**Figure 3:**
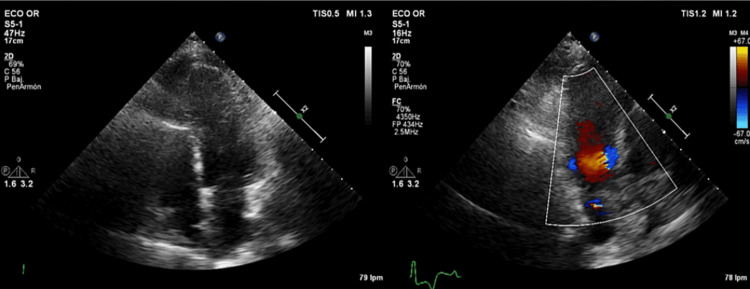
Postoperative echocardiography

Postoperative echocardiography confirmed the absence of complications, showing a heart in normal conditions and without masses (Figure 3). Blood was drawn once again, and the results came out normal. Chest X-ray showed clear lungs, with no nodules, consolidations, or collapse. The patient had a good prognosis showing significant improvement and symptom resolution. 

## Discussion

Cardiac myxomas represent the most common primary cardiac tumors, accounting for approximately 50% of all benign heart tumors. Originating primarily in the atrial chambers, their clinical presentations vary extensively, often leading to misdiagnosis or late diagnoses [16].

In the context of our case, the patient presented with progressive exertional dyspnea, a common but non-specific symptom in many cardiac and pulmonary disorders. Such manifestations in the setting of cardiac myxomas are usually attributed to the obstruction of blood flow or to embolic phenomena. Given its position and size, the tumor could impede blood flow, especially during exertion, mirroring the symptoms of conditions like mitral stenosis [17].

In the literature, there are varying presentations of cardiac myxomas depending on their size, location, and mobility. Symptoms can range from fatigue, orthopnea, and palpitations to more severe complications like syncope and sudden death [18]. The variability in presentation makes the clinical diagnosis of myxomas particularly challenging, emphasizing the role of imaging studies, especially echocardiography, as invaluable diagnostic tools. Familial occurrences of cardiac myxomas, often associated with the Carney complex, are tied to known genetic mutations [19]. However, as seen in this case and many others, the etiology behind sporadically occurring myxomas remains nebulous. 

Nonetheless, the distinction between familial and sporadic forms is critical due to the potential multi-organ involvement and recurrence in the familial form. Importantly, the treatment for cardiac myxomas is primarily surgical removal. Surgical intervention not only alleviates the symptoms but also prevents potential complications like embolization, valve obstruction, or even sudden death [3].

## Conclusions

Cardiac myxomas, constituting approximately half of all benign heart tumors, predominantly originate in the atrial chambers and present with diverse clinical manifestations, often complicating diagnosis. Our case underscores the challenge of diagnosing cardiac myxomas, as the patient's exertional dyspnea mirrored symptoms of other cardiac and pulmonary conditions. The variability in presentation, ranging from fatigue to severe complications like syncope, underscores the importance of imaging studies, particularly echocardiography, for accurate diagnosis.

While familial occurrences are associated with known genetic mutations, sporadic cases, like ours, highlight the enigmatic etiology of cardiac myxomas. Recognizing the distinction between familial and sporadic forms is crucial due to their differing implications for multi-organ involvement and recurrence. Surgical removal remains the cornerstone of treatment, addressing symptoms and averting potential complications, emphasizing the importance of prompt diagnosis and intervention in managing cardiac myxomas.
